# Genetic consequences of selection cutting on sugar maple (*Acer saccharum* Marshall)

**DOI:** 10.1111/eva.12384

**Published:** 2016-05-30

**Authors:** Noémie Graignic, Francine Tremblay, Yves Bergeron

**Affiliations:** ^1^ Institut de Recherche sur les ForêtsUniversité du Québec en Abitibi‐TémiscamingueRouyn‐NorandaQCCanada

**Keywords:** *Acer saccharum*, bottleneck, inbreeding coefficient, old‐growth forest, selection cutting, sugar maple

## Abstract

Selection cutting is a treatment that emulates tree‐by‐tree replacement for forests with uneven‐age structures. It creates small openings in large areas and often generates a more homogenous forest structure (fewer large leaving trees and defective trees) that differs from old‐growth forest. In this study, we evaluated whether this type of harvesting has an impact on genetic diversity of sugar maple (*Acer saccharum* Marshall). Genetic diversity among seedlings, saplings, and mature trees was compared between selection cut and old‐growth forest stands in Québec, Canada. We found higher observed heterozygosity and a lower inbreeding coefficient in mature trees than in younger regeneration cohorts of both forest types. We detected a recent bottleneck in all stands undergoing selection cutting. Other genetic indices of diversity (allelic richness, observed and expected heterozygosity, and rare alleles) were similar between forest types. We concluded that the effect of selection cutting on the genetic diversity of sugar maple was recent and no evidence of genetic erosion was detectable in Québec stands after one harvest. However, the cumulative effect of recurring applications of selection cutting in bottlenecked stands could lead to fixation of deleterious alleles, and this highlights the need for adopting better forest management practices.

## Introduction

Forest ecosystems are exposed to natural (i.e., fire, windstorms, pests, diseases) and human (i.e., urbanization, logging, agriculture) disturbances. The ongoing effects of climate change are being superimposed upon these disturbances in boreal (Bergeron et al. 2010), temperate (Fischer et al. 2013), and tropical (Brodie et al. 2012) forests. In northeastern North America, long‐term logging has led to changes in forest composition and structure (Boucher et al. 2009). Following logging, subsequent reductions in tree population size (tree density and forest cover) may increase genetic drift and bottlenecks and, ultimately, decrease genetic diversity (Finkeldey and Ziehe 2004). Loss of diversity may decrease the potential for a population to adapt to global changes (Hamrick 2004). Decreased genetic diversity through logging has been observed in white spruce (*Picea glauca* [Moench] Voss; Rajora 1999) and eastern white pine (*Pinus strobus* L.; Buchert et al. 1997; Rajora et al. 2000). These studies reported reductions in the mean number of alleles, low‐frequency alleles, and rare alleles, together with the level of heterozygosity. In contrast, other studies have shown no negative effects of logging on tree genetic diversity for white spruce (Fageria and Rajora 2013), black spruce (*Picea mariana* [Miller] BSP; Perry and Bousquet 2001), and black walnut (*Juglans nigra* L.; Robichaud et al. 2010). These results suggest that high intrapopulation genetic diversity, greater longevity, and efficient long‐distance pollen dispersal, which are generally observed in trees, could counterbalance and attenuate genetic losses following harvesting (Hamrick 2004).

Sugar maple (*Acer saccharum* Marshall) is a long‐lived deciduous tree that forms uneven‐aged stands. In addition to the syrup that it produces, this species has major economic value as saw timber in northeastern North America (Majcen et al. 1984; Godman et al. 1990). It is insect pollinated (bee) and wind pollinated, and it is shade tolerant (Logan 1965; Gabriel and Garrett 1984). In Canada, its range extends from southern Ontario and Québec, in the temperate deciduous forest, northwards into the boreal mixed‐wood forest (Little 1971; Saucier et al. 2003).

Recent forest management practices largely use natural disturbance dynamics to guide forest management decisions (Gauthier et al. 2009). The natural disturbance regimes of northeastern hardwoods are characterized by microgap dynamics (e.g., through the regular death and felling of single canopy trees or small groups of trees). Since the early 1990s, single‐tree selection cutting, a type of partial cut that is well‐adapted to the uneven‐age structures of northeastern hardwood forests, has become the most common harvesting treatment for sugar maple stands in Québec (Majcen 1994). It was implemented to replace the diameter‐cut limit, which is a sylvicultural system that consists of removing only large merchantable timber trees, while leaving poor‐quality trees (Majcen 1994). Selection cutting is believed to better emulate the small‐scale gap disturbance dynamics of sugar maple stands. It consists in the removal of 25–35% of the volume of trees having a diameter at breast height (d.b.h.) ≥10 cm (MRNFPQ 2003). The single‐tree is selected in different diameter classes, with cutting cycles occurring at regular intervals of 15–25 years to sustain stand structure and maintain its quality (MRNFPQ 2003). Selection cutting should not be confused with selective cutting (or selective logging), which is a practice that targets the removal of the largest or most marketable timber while, in many cases, leaving poor‐quality trees standing without necessary regard for the future of the stands.

Stands that are harvested using single‐tree selection have a smaller number of large and defective trees than do old‐growth stands (Angers et al. 2005). Fifteen years after an experimental selection cut in sugar maple stands, the basal area, radial growth, and development of sugar maple, American beech (*Fagus grandifolia* Ehrhart) and yellow birch (*Betula alleghaniensis* Britton) saplings were higher than those within control plots (Majcen et al. 2005). However, commercially harvested hardwood forests that were treated with selection cuts in Québec were 55% less productive after 10 years, in terms of growth of usable timber compared to experimental plots that had been surveyed (Guillemette et al. 2013). This response is possibly due to the low initial (preharvest) quality of sugar maple stands or to higher intensity harvesting of good quality stems (Guillemette et al. 2013).

Rainville (2007) noted that basic knowledge of hardwood tree species genetic diversity across Canada is lacking. Previous work on the genetic diversity of sugar maple is scarce and has focused mainly on the effects of fragmentation incurred by agriculture and clear‐cutting. Results of these studies are not consistent. Foré et al. (1992b) reported higher genetic differentiation among fragmented patches for sugar maples canopy trees than juveniles. Foré et al. (1992b) concluded that the level of gene flow between forest patches was higher in this open landscape. Both Foré et al. (1992a) and Baucom et al. (2005) found no differences between sugar maple cohorts (juvenile versus adults) within patches. In comparing the genetic diversity of 1‐year‐old seedlings between patches and continuous forest, Young et al. (1993a) and Young and Merriam (1994) found greater genetic diversity within the fragmented forest. Eighty years after one clear‐cut, sugar maple seedlings had a lower percentage of polymorphic loci and allelic richness estimates compared to an old‐growth forest (Baucom et al. 2005).

Selection cutting is a treatment that emulates tree‐by‐tree replacement in forests with uneven‐age structures. However, it creates small openings in large areas of canopy and often generates forest structures that differ markedly from old‐growth forest. The fact that selection cutting, as the state‐of‐the‐art sustainable forestry practice, may potentially harm forest genetics has not been taken into consideration. In this study, we evaluated whether selection cutting has an effect on sugar maple genetic diversity. We studied this type of harvesting because selection cutting is the most commonly applied harvesting treatment in the northeastern hardwood forest, and there have been no studies of its potential impacts on sugar maple genetic diversity. It is important to address this question in a context where international forest certification processes (e.g., the Forest Stewardship Council) are promoting sustainable forest management practices that include the protection of genetic diversity of forest trees. Using highly polymorphic microsatellite markers (Graignic et al. 2013), we compared for the first time the genetic structure of mature and regeneration sugar maple cohorts in logged stands after selection cutting to that of adjacent unlogged stands (old growth). We hypothesized that: (i) the removal of 30% (by volume) of mature trees would reduce the level of diversity (mean number of alleles, rare alleles, and the level of heterozygosity) of the mature sugar maple cohort compared to the old‐growth mature sugar maple cohort; (ii) sugar maple seedlings, which had established after logging, would have a lower level of genetic diversity due to reductions in the number of potential parent trees (mature trees); and (iii) selection cutting would influence levels of genetic structure among cohorts (adults–saplings–seedlings).

## Materials and methods

### Study area and sampling

**Figure 1:**
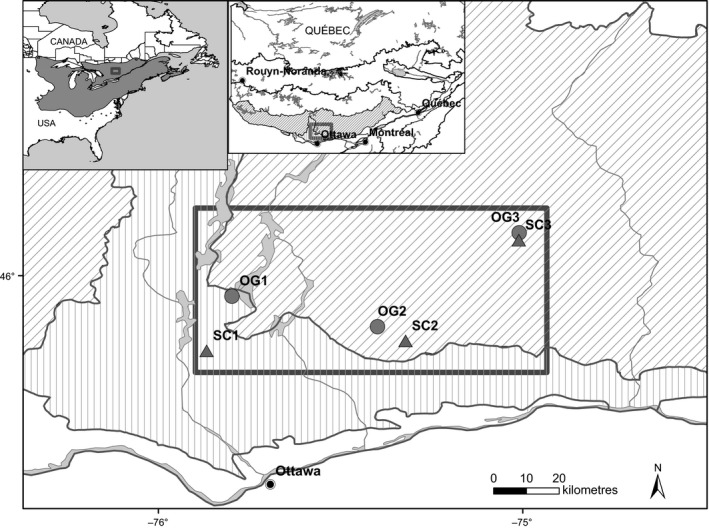
Map of the study area, which is situated at the northern continuous distributional limits of sugar maple (*Acer saccharum*) in Québec, showing the locations of the 6 study sites (circles, old‐growth sites; triangles, selection cutting sites), sugar maple–yellow birch (*Betula alleghaniensis*) bioclimatic domain (slanted hatching), sugar maple–basswood (*Tilia americana*) bioclimatic domain (vertical hatching), and boundaries of all bioclimatic domain limits (thin lines) (Saucier et al. 2003).

The study took place in the continuous portion of the northern range of sugar maple, that is, southern Québec, Canada (Fig. 1). The study area was located between 45°46′N and 46°8′N, and 75°53′W and 74°59′W, at elevations ranging between 305 and 410 m (Table S1). This zone lies within the sugar maple–yellow birch (*A. saccharum*–*B. alleghaniensis*) and sugar maple–basswood (*A. saccharum*–*Tilia americana* L.) bioclimatic domains, where sugar maple is abundant (Saucier et al. 2003).

The stands that had been subjected to selection cutting were harvested once, during winter 1990 (at the end of 1990 and early 1991). The selection cutting (SC) stands were part of a commercial harvest that consisted of the removal of 25–35% of tree basal area every 15–25 years (MRNFPQ 2003). The old‐growth (OG) stands, which had not been logged, were classified as exceptional forest ecosystems (EFE) by the *ministère des Ressources naturelles du Québec* (Québec Ministry of Natural Resources; (MRNQ 2013). The logged stands were paired with adjacent unlogged stands. One pair of sites (OG1 and SC1) was located in the sugar maple–basswood bioclimatic domain, while the other two were in the sugar maple–yellow birch bioclimatic domain (Fig. 1). All six sites, except for OG2, were similar to those described by Angers et al. (2005), and OG2 and OG3 are similar to 2‐C‐D and 2‐C‐C described by Graignic et al. (2014). The respective distances between logged and unlogged sites were 17.3 km (OG1‐SC1), 7.8 km (OG2‐SC2), and 1.6 km (OG3‐SC3) (Table S1).

Tissue samples (usually leaves or bark) from 360 sugar maple individuals were collected in 2008. Three cohorts were sampled randomly per site: mature trees (M, ≥10 cm d.b.h., *n *=* *20–22), saplings (Sa, 1 ≤ d.b.h. <10 cm, *n *=* *20), and seedlings (S, d.b.h. <1 cm, *n *=* *18–20). Seedling age was determined as described by Graignic et al. (2014), with seedlings corresponding to seed that had been produced between 1986 and 2006. Most of the oldest seedlings originated from a mast event that had occurred in 1996 (Graignic et al. 2014). This cohort could have been directly affected by reductions in the number of potential parent trees in SC. Samples were dried over silica gel and maintained at room temperature until they were needed for genetic analyses.

### Molecular methods

DNA was extracted using Extract‐N‐Amp^™^ Plant PCR Kits (Sigma‐Aldrich, Oakville, ON, Canada). All samples were genotyped for 18 variable microsatellite loci using PCR and genotyping protocols as previously reported (Graignic et al. 2013). The following modifications to the protocol were applied: (i) five different multiplex PCR sets were used (see Table S2); (ii) 0.1 μm of each primer; and (iii) 37 cycles in the PCR amplification procedure.

### Marker genetic diversity

For each locus, the total number of alleles (*A*_T_), mean number of alleles per locus (*A*), mean observed (*H*_O_) and expected (*H*_E_) heterozygosity, and the inbreeding coefficient (*F*_IS_) were estimated using fstat 2.9.3.2 (Goudet 2001). Departure from Hardy–Weinberg equilibrium (HWE) per locus in each stand was tested, together with linkage equilibrium between all pairs of loci in each stand, were conducted using exact tests in genepop 4.2.1 (Rousset 2008). Markov chain parameters for HWE were 10 000 dememorizations, followed by 500 batches of 5000 iterations per batch. We corrected for multiple comparisons using sequential Bonferroni adjustment of *P*‐values to a predetermined experiment‐wise error rate of 0.05 (Rice 1989). Null allele frequencies were estimated using freena (10 000 replicates; Chapuis and Estoup 2007). This program was selected because it uses the algorithm of Dempster et al. (1977), which provided the most accurate estimates among the several algorithms that were tested by Chapuis and Estoup (2007). We performed a Mantel test (1000 permutations) between pairwise *F*_ST_ values, with and without correction for null alleles that were calculated with freena. These tests were performed using the *mantel* function in the vegan library (Oksanen et al. 2011) within the R statistical environment (version 2.13.1, R Development Core Team 2011).

### Genetic diversity and differentiation between cohorts and forest types

For each stand, the mean number of alleles per locus (*A*), mean allelic richness (*A*_R_), mean observed heterozygosity (*H*_O_), mean expected heterozygosity (*H*_E_), pairwise *F*_ST_, mean pairwise *F*_ST_, and inbreeding coefficients (*F*_IS_) were estimated using fstat. Tests for heterozygote deficiency were performed using genepop (Markov chain parameters: 10 000 dememorizations, followed by 500 batches of 5000 iterations per batch; Fisher's exact tests). Tests were calculated for each stand using data for pooled individuals (PI), mature trees (M), saplings (Sa), and seedlings (S), separately. The genetic analysis was conducted using all the markers and also markers with < 10 alleles per locus. According to Balloux et al. (2000), when the population size is small and markers are highly variable, there are possibilities that these markers may not capture all alleles that are present in the stand, which might yield misleading results. Allelic richness was estimated for each cohort and sampled sites using a rarefaction method (El Mousadik and Petit 1996).

To compare *A*_R_, *H*_O_, *H*e, and *F*_IS_ between forest types (OG and SC) and between cohorts (M, Sa, and S), we performed a linear mixed‐model analysis (LMM, using the *lme* function in the nlme library of R; Pinheiro et al. 2011). The fixed effects were forest type and cohort and their two‐way interactions; microsatellite marker and pair were considered as random effects. Assumptions of normality and homoscedasticity were verified graphically. Models were simplified by stepwise backward elimination of nonsignificant fixed effects terms to produce the most parsimonious model. There were few significant values of pairwise *F*_ST,_ and this index was subsequently excluded from the analysis. The same indices, *(A*_R_, *H*_O_, *H*e, and *F*_IS_) were also compared between forest types and cohorts using fstat (1000 permutations).

### Partitioning of molecular variation

The structure of the genetic variation was determined using the hierarchical analysis of molecular variance (amova) that is implemented in genalex 6.5b3 (Peakall and Smouse 2006). Genetic differentiation among populations was estimated by the Φ_PT_ statistic. We performed separate significance tests (9 999 permutations) between pairs (1, 2, and 3), between forest types (OG and SC) within pairs and within stands, and between pairs and between cohorts (M, Sa, and S) within pairs and within cohorts for OG and SC.

### Allele frequencies

The effect of selection cutting on allele frequencies was tested using four classes: common (*f *≥* *0.75), intermediate (0.75 >  *f *≥* *0.25), low (0.25 >  *f *≥* *0.01), and rare (*f *<* *0.01). We also added two classes, low (0.25 >  *f *≥* *0.05) and rare (*f *<* *0.05), using a level of 0.05, as suggested by Marshall and Brown (1975). Allele frequencies were estimated using fstat. The analyses were performed for each cohorts (M, Sa, and S) and cohort within forest types.

### Bottlenecks

To test for recent reductions in effective population size following selection cutting, we used bottleneck 1.2.02 (Cornuet and Luikart 1996) for each stand and cohort within each stand. Evidence of bottlenecks was tested using heterozygote excess and allele frequency mode‐shift tests. For the test of heterozygote excess, we used three different mutation models: infinite allele mutation (IAM), stepwise‐mutation model (SMM), and two‐phase mutational model (TPM). In TPM, we chose 70%, 90%, 95%, and 99% SMM, and 12% variance of multistep mutations was assumed (Piry et al. 1999). Significance tests used one‐tailed Wilcoxon signed‐rank tests. Population bottlenecks cause a mode‐shift distortion of the typical L‐shaped allele frequency distribution (Luikart and Cornuet 1998). We also used the graphical method to assess a bottleneck‐induced distortion of allele frequency distributions that cause alleles at low frequency (<0.025) to become less abundant than alleles in one or more allele higher frequency classes (e.g., >0.025–0.050) (Luikart et al. 1998).

## Results

### Genetic variability of microsatellite markers

The total number of alleles per locus ranged from 3 to 28, while the mean number of alleles per locus ranged from 2.8 to 17.7 (Table S2). Five of the 18 loci (SM22, SM27, SM47, SM55, and SM56) failed to meet HWE in three populations (Table S3). These deviations from HWE were due to heterozygote deficiencies (*F*_IS_ ≥0.296; Table S2). Four other markers also showed heterozygote deficiencies (*F*_IS_ ≥0.246 for SM29, SM51, SM53, and SM60). These loci exhibited higher frequencies of null alleles (≥0.10) (Table S4). Their genetic structures (pairwise *F*_ST_) were similar before and after correction for the presence of null alleles (*r *=* *0.90, *P *=* *0.003). All loci were considered independent because no significant linkage disequilibrium between pairs of loci within forest types was detected after Bonferroni correction. All loci (18) were used in further analyses.

### Genetic diversity and differentiation between cohorts and forest types

The mean number of alleles per locus (*A*) ranged from 9.2 (SC3) to 9.5 (SC1, OG2, SC2, and OG3) for stands and from 6.5 (M in SC2 and M in SC3) to 7.6 (M in OG3) for cohorts (Table 1). No significant difference was detected between cohorts and forest types in terms of allelic richness (*A*_R_) and expected heterozygosity (*H*_E_), using both LMM (in R) and fstat (Tables S5 and S6).

**Table 1:** Genetic variability estimates of sugar maple (*Acer saccharum*) stands in Outaouais, Québec, for mature trees (M), saplings (Sa), seedlings (S), and pooled individuals (PI)

Stands	Cohorts	*n*	*A*	*A*_R_ †	*H*_O_	*H*_E_	*F*_IS_	*F*_ST_
OG1	M	20	7.1	6.4	0.583	0.692	0.158***	0.003
	Sa	20	6.6	6.0	0.440	0.676	0.348***	0.000
	S	20	7.1	6.4	0.496	0.684	0.274***	0.004
	PI	60	9.4	9.0	0.507	0.686	0.260***	0.000
SC1	M	20	7.2	6.4	0.583	0.706	0.174***	0.001
	Sa	20	6.9	6.2	0.480	0.697	0.312***	0.006
	S	20	6.9	6.3	0.521	0.690	0.245***	0.003
	PI	60	9.5	9.1	0.529	0.699	0.243***	0.001
OG2	M	20	6.9	6.3	0.617	0.696	0.114***	0.001
	Sa	20	6.9	6.3	0.520	0.676	0.230***	0.003
	S	20	7.0	6.3	0.516	0.693	0.255***	0.002
	PI	60	9.5	9.0	0.552	0.688	0.198***	−0.001
SC2	M	20	6.5	6.2	0.597	0.698	0.145***	0.005
	Sa	20	6.7	6.1	0.498	0.658	0.242***	0.003
	S	20	7.0	6.4	0.539	0.712	0.243***	0.007
	PI	60	9.5	9.1	0.543	0.694	0.218***	0.000
OG3	M	22	7.6	6.6	0.629	0.695	0.095*	0.002
	Sa	20	6.7	6.1	0.544	0.688	0.209***	0.005
	S	18	6.8	6.3	0.548	0.687	0.202***	0.008
	PI	60	9.5	9.0	0.577	0.693	0.168***	0.002
SC3	M	20	6.5	5.9	0.553	0.690	0.199***	0.001
	Sa	20	7.4	6.7	0.531	0.698	0.239***	0.003
	S	20	6.8	6.1	0.452	0.683	0.337***	0.008
	PI	60	9.2	8.8	0.511	0.690	0.260***	0.001
Means			9.4	9.0	0.536	0.692	0.225***	0.000
All			13.5	13.2	0.537	0.692	0.224***	

*n*, number of individuals; *A*, mean number of alleles per locus; *A*_R_, mean allelic richness; *H*_O_, mean observed heterozygosity; *H*_E_, mean expected heterozygosity; *F*_IS_, inbreeding coefficient; *F*_ST_, mean pairwise *F*_ST_; Means were determined using PI. All individuals were included in tests for heterozygote deficiency: ***P ≤* *0.001; *0.005 < *P *≤* *0.010.

†Allelic richness was estimated with *n* = 18 for each cohort (M, Sa, and S) and *n* = 60 for PI. Populations were old‐growth (OG) forest or were subjected to a single selection cutting (SC) at the end of 1990–beginning of 1991.

**Figure 2:**
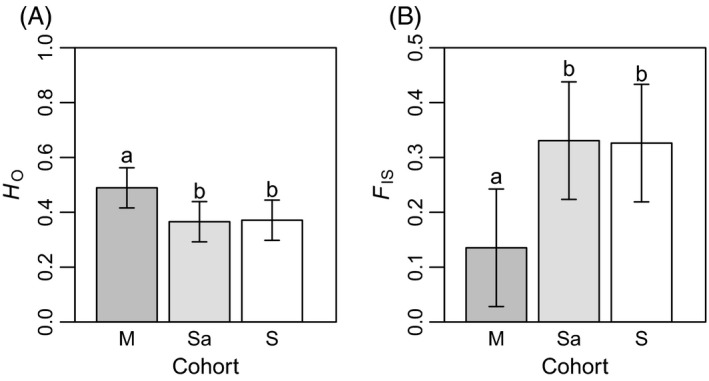
Predicted means (95% confidence intervals) of *H*_O_ (A) and *F*_IS_ (B) for cohorts (M: mature sugar maples, Sa: sugar maple saplings, and S: sugar maple seedlings). *H*_O_, mean observed heterozygosity; *F*_IS_, inbreeding coefficient. Means with the same letter do not differ at *α *= 0.05, but differ (with an asterisk) at *α *= 0.10.

Observed heterozygosity (*H*_O_) and the inbreeding coefficient (*F*_IS_) ranged from 0.440 (Sa in OG1) to 0.629 (M in OG3) and from 0.095 (M in OG3) to 0.348 (Sa in OG1), respectively (Table 1). All cohorts of all populations had a significant heterozygote deficit (*P*‐values < 0.0054), suggesting a departure from random mating in both forest types (Table 1). We found a significant difference between cohorts for both forest types with higher mean *H*_O_ and lower mean *F*_IS_ for mature trees (*H*_O_ = 0.594; *F*_IS_ = 0.144) compared to saplings (*H*_O_ = 0.505; *F*_IS_ = 0.269) and seedlings (*H*_O_ = 0.512; *F*_IS_ = 0.259) (Table S6, Fig. 2). Similar results were obtained using fstat (Table S5).

Means of *F*_ST_ between stands were very low (−0.001−0.002; Table 1). Only 3 pairwise *F*_ST_ were significant (OG3‐SC1, OG3‐SC3, and SC1‐SC3), but their respective values were very low (0.004, 0.004, and 0.002; Table S7).

Similar results were obtained when only the less variable markers (≤10 alleles/locus) were included in the analysis (Tables S5–S8, Figure S1). However, as more significant pairwise *F*_ST_ values were detected using all markers (Table S7), subsequent analyses were performed with all markers included.

### Partitioning of molecular variation

amova revealed that most of the variation (99–100%, *P *=* *0.015–0.176; Table 2) was attributable to genetic variation within groups (stands or cohorts). A slight effect of cohorts (1%, *P *=* *0.034; Table 2) was detected in the SC stands.

**Table 2:** Results of analysis of molecular variance (amova) showing the partitioning of genetic variance among pairs, forest types, and cohorts

Source of variation	df	Sums‐ of‐squares	Variance components	Percentage of variance	Phi (Φ) statistics	*P*‐values
Among pairs	2	36.397	0.000	0	0.000	0.620
Between forest types within pairs	3	57.125	0.042	0	0.003	*0.070*
Within stands	354	5858.567	16.550	100	0.002	*0.062*
Total	359	5952.089	16.591	100		
OG						
Among pairs	2	34.050	0.000	0	0.000	0.476
Between cohorts within pairs	6	101.998	0.047	0	0.003	0.205
Within cohorts	171	2746.652	16.062	100	0.003	0.176
Total	179	2882.700	16.110	100		
SC						
Among pairs	2	39.261	0.006	0	0.000	0.416
Between cohorts within pairs	6	115.517	0.116	1	0.007	**0.034**
Within cohorts	171	2894.400	16.926	99	0.007	**0.015**
Total	179	3049.178	17.049	100		

df, degrees of freedom. Significant values at *α *= 0.05 are in bold type and at *α *= 0.10 in italics.

### Allele frequencies

No difference for allele frequencies was detected between selection cutting versus unlogged stands and among cohorts (PI, M, Sa, and S) (Tables 3 and S9).

**Table 3:** Number of alleles per classes of frequency, per cohort of the populations, and grouping populations by forest types

Forest types	Cohorts	*A*_T_	C	I	L0.01	R0.01	L0.05	R0.05
OG	M	179	0 (0%)	22 (12%)	116 (65%)	41 (23%)	55 (31%)	102 (57%)
	Sa	165	1 (1%)	21 (13%)	103 (62%)	40 (24%)	57 (35%)	86 (52%)
	S	174	1 (1%)	20 (11%)	121 (70%)	32 (18%)	53 (30%)	100 (57%)
	PI	217	0 (0%)	21 (10%)	128 (59%)	68 (31%)	53 (24%)	143 (66%)
SC	M	167	0 (0%)	24 (14%)	110 (66%)	33 (20%)	53 (32%)	90 (54%)
	Sa	171	1 (1%)	18 (11%)	116 (68%)	36 (21%)	60 (35%)	92 (54%)
	S	175	0 (0%)	20 (11%)	118 (67%)	37 (21%)	57 (33%)	98 (56%)
	PI	215	1 (0%)	18 (8%)	127 (59%)	69 (32%)	61 (28%)	135 (63%)
All	M	202	0 (0%)	21 (10%)	126 (62%)	55 (27%)	56 (28%)	125 (62%)
	Sa	198	1 (1%)	20 (10%)	121 (61%)	56 (28%)	55 (28%)	122 (62%)
	S	207	0 (0%)	21 (10%)	125 (60%)	61 (29%)	52 (25%)	134 (65%)
	PI	243	0 (0%)	19 (8%)	57 (23%)	167 (69%)	129 (53%)	95 (39%)

*A*_T_, total number of alleles; C, common *f *≥* *0.75; I, intermediate 0.75 > *f *≥* *0.25; L0.01, low 0.25 > *f *≥* *0.01; R0.01, rare *f *<* *0.01; L0.05, low 0.25 > *f *≥* *0.05; R0.05, rare *f *<* *0.05; M, mature sugar maples; Sa, sugar maple saplings; S, sugar maple seedlings; PI, pooled individuals.

### Bottlenecks

**Figure 3:**
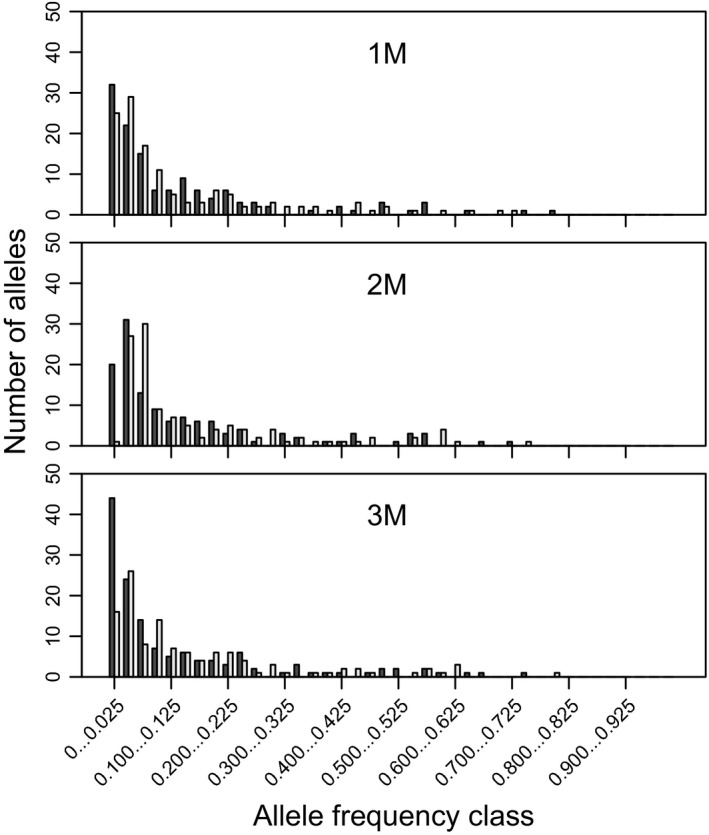
Allele frequency distributions from old‐growth stands (black bars) and selection cutting stands (open bars). 1M, mature sugar maples from OG1 and SC1; 2M, mature sugar maples from OG2 and SC2; and 3M, mature sugar maples from OG3 and SC3.

Recent bottlenecks were detected in the three SC stands, while none were apparent in the OG stands using IAM (Table 4). In SC stands, recent bottlenecks were detected in the mature cohorts (Table S10). Yet, no bottleneck was detected in any stand using SMM, TMM, and mode‐shift models (Tables 4 and S10). The graphical method revealed an allelic pattern that was typical of a recent bottleneck in mature sugar maple cohorts of the three SC stands, with fewer alleles detected in the low‐frequency class (<0.025) than in intermediate frequency classes (e.g., >0.025–0.050) (Figure S2). Conversely, nonbottlenecked OG stands had an allele frequency distribution with a mode in the low‐frequency class (Figs 3 and S3).

**Table 4:** Bottlenecks results based on heterozygosity excess and mode shift

Stands	Heterozygosity excess						Mode shift
	IAM	TMM				SMM	
		70%	90%	95%	99%		
OG1	0.08368	0.97003	0.99961	0.99994	1.00000	1.00000	Normal
SC1	**0.04488**	0.98075	0.99671	0.99883	0.99968	0.99974	Normal
OG2	0.06487	0.98658	0.99968	0.99992	0.99999	0.99999	Normal
SC2	**0.04071**	0.99552	0.99983	0.99995	0.99995	0.99997	Normal
OG3	0.05935	0.97842	0.99480	0.99832	0.99903	0.99961	Normal
SC3	**0.00140**	0.99800	0.99995	0.99999	1.00000	1.00000	Normal

Significant values (*α *= 0.05) are in bold type.

## Discussion

### Sugar maple genetic diversity

We observed a high level of genetic diversity in sugar maple. Previous studies using allozymes have also shown high levels of genetic diversity in this species (Table 5; *A *=* *2.3, range: 1.10–5.00; *H*_E_ = 0.131, range: 0.015–0.275), which is typical of *Acer* species and other tree species in northeastern North America (Table 5). Similar levels of diversity were reported for Norway maple (*Acer platanoides* L.), bigleaf maple (*A. macrophyllum* Pursh), American chestnut (*Castanea dentata* [Marsh.] Borkh.), and red oak (*Quercus rubra* L.), while lower levels have been observed in sycamore maple (*A. pseudoplatanus* L.) (Table 5). We found slightly higher values for *A* and *A*_R_ than have been previously reported using the same 18 microsatellites (Table 5; Graignic 2014), which was likely because the number of individuals that were sampled per site was higher here (*n *=* *60 vs *n *=* *40). Diversity was found principally within stands in both studies (Table 2 and Graignic 2014). Values for *A*,* A*_R,_ and *H*_E_ were higher than or similar to those reported for other *Acer* species and many other tree species in northeastern North America (Table 5).

**Table 5:** Comparison of genetic diversity using allozyme and microsatellite markers for populations of tree species in northeastern North America, and *Acer* species worldwide

Species	*A*	*A*_R_	*H*_O_	*H*_E_	*F*_IS_	Reference
Allozymes						
Angiosperm						
*Acer platanoides*	1.92 (1.50–2.50)	—	0.129 (0.066–0.238)	0.128 (0.063–0.207)	−0.012 (−0.373–0.189)	Rusanen et al. (2000)
*Acer platanoides*	2.0 (1.6–2.4)	—	0.126 (0.038–0.195)	0.132 (0.053–0.191)	0.066 (−0.085–0.285)	Rusanen et al. (2003)
*Acer pseudoplatanus*	2.78 (2.56–3.00)	—	0.293 (0.237–0.327)	0.238 (0.254–0.319)	−0.032 (−0.159–0.085)	Belletti et al. (2007)
*Acer macrophyllum*	1.71 (1.5–2.2)	—	0.118 (0.102–0.160)	0.152 (0.102–0.189)	0.166 (−0.086–0.332)	Iddrisu and Ritland (2004)
*Acer saccharum*	1.95 (1.64–2.18)	—	—	0.110 (0.098–0.132)	—	Perry and Knowles (1989)
*Acer saccharum*	2.2 (1.1–2.8)	—	0.169 (0.08–0.28)	0.171 (0.08–0.29)	—	Foré et al. (1992b)
*Acer saccharum*	2.9	—	0.15	0.148	—	Foré et al. (1992a)
*Acer saccharum*	2.07 (2.03–2.10)	—	—	0.115 (0.109–0.121)	0.062 (0.050–0.073)	Young et al. (1993a)
*Acer saccharum*	1.98 (1.78–2.41)	—	—	0.112 (0.088–0.138)	0.042 (−0.095–0.177)	Young et al. (1993b)
*Acer saccharum*	1.83	—	0.136	0.148	0.077	Simon et al. (1995)
*Acer saccharum*	3.46 (2.00–5.00)	1.99 (1.14–2.98)	0.113 (0.021–0.294)	0.116 (0.015–0.275)	0.025 (−0.108–0.073)	Baucom et al. (2005)
*Castanea dentata*	1.69 (1.50–1.89)	—	0.184 (0.135–0.264)	0.151 (0.096–0.196)	−0.226	Huang et al. (1998)
*Fagus grandifolia*	2.9 (2.9–2.9)	—	0.387 (0.382–0.392)	0.395 (0.383–0.407)	0.024	Houston and Houston (1994)
*Fagus grandifolia*	3.0 (2.78–3.33)	—	0.163 (0.150–0.175)	0.165 (0.150–0.179)	—	Houston and Houston (2000)
*Populus tremuloides*	2.7 (2.1–2.9)	—	0.125 (0.101–0.160)	0.235 (0.207–0.270)	0.462 (0.295–0.568)	Hyun et al. (1987)
*Populus tremuloides*	2.6 (2.2–2.9)	—	0.217 (0.197–0.242)	0.220 (0.193–0.244)	0.017	Lund et al. (1992)
*Quercus rubra*	2.08 (1.8–2.3)	—	—	0.186 (0.145–0.245)	0.100	Sork et al. (1993)
Gymnosperm						
*Picea glauca*	3.03 (2.17–3.83)	2.14 (1.86–2.37)	0.342 (0.221–0.414)	0.344 (0.199–0.412)	0.002 (−0.092–0.087)	O'Connell et al. (2006)
*Picea rubens*	1.47 (1.25–1.64)	—	0.075 (0.059–0.092)	0.079 (0.061–0.104)	0.043 (−0.037–0.224)	Hawley and DeHayes (1994)
*Pinus strobus*	2.02 (1.69–2.37)	—	0.129 (0.121–0.143)	0.152 (0.146–0.157)	—	Buchert et al. (1997)
*Pinus strobus*	2.35 (2.23–2.50)	—	0.215 (0.185–0.216)	0.195 (0.181–0.216)	−0.090 (−0.200–0.053)	Rajora et al. (1998)
*Thuja occidentalis*	1.6 (1.5–1.8)	—	0.116 (0.102–0.133)	0.129 (0.113–0.141)	0.106	Lamy et al. (1999)
Microsatellites						
Angiosperm						
*Acer mono*	12.63	—	—	0.802	−0.008	Kikuchi et al. (2009)
*Acer mono*	—	8.37 (7.38–9.65)	—	0.80 (0.70–0.85)	0.27 (0.20–0.32)	Takayama et al. (2012)
*Acer okamotoanum*	—	6.60 (6.11–7.41)	—	0.72 (0.66–0.76)	0.18 (0.03–0.24)	Takayama et al. (2012)
*Acer pseudoplatanus*	—		0.548 (0.543–0.553)	0.574 (0.573–0.574)		Pandey (2005)
*Acer pseudosieboldianum*	—	4.60 (3.79–5.25)	0.40 (0.32–0.46)	0.61 (0.53–0.68)	0.33 (0.21–0.43)	Takayama et al. (2013)
*Acer saccharum*	9.4 (9.2–9.5)	9.0 (8.8–9.1)	0.536 (0.507–0.577)	0.692 (0.686–0.699)	0.225 (0.168–0.260)	Our study
*Acer saccharum*	8.2 (6.6–9.0)	7.0 (5.8–7.6)	0.597 (0.496–0.716)	0.693 (0.637–0.715)	0.138 (−0.051–0.302)	Graignic (2014)
*Acer skutchii*	2.1 (1.5–2.5)	—	—	0.129 (0.054–0.247)	—	Lara‐Gomez et al. (2005)
*Acer takesimense*	—	3.82 (3.59–4.23)	0.38 (0.30–0.47)	0.53 (0.48–0.58)	0.28 (0.08–0.47)	Takayama et al. (2013)
*Quercus ellipsoidallis*	13	—	0.67 (0.62–0.72)	0.79 (0.77–0.81)	0.145 (0.10–0.19)	Lind and Gailing (2013)
*Quercus rubra*	14.5 (13–15)	—	0.73 (0.70–0.75)	0.84 (0.83–0.86)	0.12 (0.07–0.17)	Lind and Gailing (2013)
*Populus tremuloides*	8.83 (7.58–10.08)	—	0.465 (0.45–0.48)	0.67 (0.61–0.73)	0.30 (0.21–0.39)	Namroud et al. (2005)
*Populus tremuloides*		5.99 (3.34–6.83)		0.758 (0.613–0.801)	0.019 (−0.12–0.19)	Callahan et al. (2013)
*Populus tremuloides*	7.44 (6.25–8.2()	—	0.556 (0.478–0.704)	0.725 (0.691–0.767)	0.201 (−0.054–0.325)	Wyman et al. (2003)
Gymnosperm						
*Pinus strobus*	8.21 (6.85–9.62)	—	0.516 (0.485–0.538)	0.597 (0.585–0.615)	—	Rajora et al. (2000)
*Pinus strobus*	7.7	7.0 (6.7–7.3)	0.465 (0.46–0.47)	0.485 (0.48–0.49)	0.03 (0.01–0.05)	Marquardt and Epperson (2004)
*Thuja occidentalis*	9.58 (7.83–11.17)	9.21 (7.66–10.68)	0.590 (0.505–0.640)	0.600 (0.519–0.662)	0.019 (−0.025–0.050)	Pandey and Rajora (2012)
*Thuja occidentalis*	7.8 (5.0–10.0)	5.9 (4.6–6.9)	0.734 (0.463–0.883)	0.773 (0.712–0.840)	0.145	Xu et al. (2012)

*A*, mean number of alleles per locus; *A*_R_, mean allelic richness; *H*_O_, mean observed heterozygosity; *H*_E_, mean expected heterozygosity; *F*_IS_, inbreeding coefficient. Range values were in parenthesis. ‘—’ data were not available.

We observed a positive and significant inbreeding coefficient (*F*_IS _= 0.225, range: 0.168–0.260). The presence of heterozygote deficiency is common in trees and has been reported for *Acer* species and trembling aspen (*Populus tremuloides* Michaux) using microsatellite markers (Table 5). Heterozygote deficiency is often associated with inbreeding (high levels of consanguineous mating) or is due to a Wahlund effect (mixing of differentiated gene pools). However, sugar maple is recognized for its low self‐compatibility, which is related to dichogamy (Gabriel 1968), together with its frequent postfertilization ovule abortion following self‐pollination (Gabriel and Garrett 1967). In the present study, sugar maple stands were localized within a small area relative to the species range and *F*_IS_ values can vary widely among populations (Table 5 and Graignic 2014). Significant variation in *F*_IS_ (heterozygote excess to deficiency) has been reported for trembling aspen and Korean maple (*A. takesimense* Nakai), across their natural geographic ranges (Table 5; Namroud et al. 2005; Callahan et al. 2013; Takayama et al. 2013). Thus, our *F*_IS_ values likely reflected the level of genetic diversity that we observed for sugar maple in our study area.

### Cohort differences

Lower observed heterozygosity (*H*_O_) and higher *F*_IS_ was observed in the younger cohorts (Sa, S) compared to mature sugar maple trees (Fig. 2). The level of heterozygosity increased with age (here, in trees with d.b.h. ≥10 cm) in the SC and OG stands, a result that is similar to other studies that have compared cohorts of different ages. This pattern has been reported in various coniferous species (Bush and Smouse 1992; Nijensohn et al. 2005), in dragon spruce (*Picea asperata* Masters; Wang et al. 2010), and in sugar maple (Ballal 1994). However, this pattern is not always observed in sugar maple, because other studies found no differences among cohorts (Foré et al. 1992a; Graignic 2014). One explanation for the presence of a higher level of heterozygosity in mature trees is selection against homozygotes that occurred during the self‐thinning process. This may be explained by higher fitness of heterozygote individuals or the reduced survival of offspring of related individuals (Charlesworth and Willis 2009). Sugar maple typically forms uneven‐aged stands and the mortality rate at the seedling stage is very high, particularly for younger seedlings (Graignic et al. 2014). In theory, we would expect a decrease in the low‐frequency allele class with age because during selection, rare deleterious alleles would be eliminated (Charlesworth and Willis 2009). In fact, however, the percentage of rare alleles was similar among mature sugar maple, saplings, and seedlings (Table 3).

Another possible explanation is that mature sugar maple trees originated from overlapping generations, given that sugar maple can live from 300 to 400 years (Godman et al. 1990). The d.b.h. of mature sugar maple trees that were sampled ranged between 10 and 82 cm, which corresponds to ages between 35 and 285 years (Majcen et al. 1984; Graignic et al. 2014). These generations could have been influenced by different random selection processes, both at their establishment and throughout their life spans. In contrast, the seedling populations originated from four seed masts, mostly from a mast event that occurred in 1996 (Graignic et al. 2014). The same selection processes have not visibly influenced those very few generations of seedlings, given the short period of influence (around 12 years) compared to mature sugar maple trees (285 years).

Contrary to our expectations, the level of genetic structure between cohorts was similar among forest types. At the stand level, no significant impacts on allelic richness and genetic diversity (*H*_E_) of the regeneration cohorts (seedlings, saplings) were observed in the selection cutting stands. Similar results have been obtained in other tree species. For example, fragmentation (clear‐cutting) did not change the level of genetic diversity in seedling populations of mountain hemlock (*Tsuga mertensiana* [Bong.] Carrière), relative to both adults and seedlings in old‐growth forests (Ally and Ritland 2007). No strong effects of small and low‐density forests on the genetic diversity of naturally established seedling cohorts were likewise detected in pedunculate oak (*Quercus robur* L.) (Vranckx et al. 2014). In the present study, mean pairwise relatedness estimates (*r*) in sugar maple (both for adult and seedling cohorts) were very low (range: −0.001 ≥ *r *>* *0.005; data not shown), which were indicative of unrelated individuals. It is plausible that extensive gene flow occurring between sugar maple populations had compensated for reductions in the number of potential parent trees (mature trees) within selection cutting stands and helped maintain a high level of diversity in seedling cohorts that have regenerated after harvesting. Khodwekar et al. (2015) reported that 78–82% of gene flow in a sugar maple stand originates from outside of the stand. These authors also established the absence of fine‐scale spatial genetic structure (SGS), suggesting effective dispersal of both seeds and pollen.

### Selection cutting influences and implications

We detected a significant deviation from mutation‐drift equilibrium under IAM in the three harvested stands, but not in the three old‐growth stands. This response was particularly noticeable for mature sugar maples (Tables 4 and S10). IAM was better at detecting subtle genetic bottlenecks; microsatellites that are 2‐bp repeat units in length were best modeled by IAM (López‐Flores and Garrido‐Ramos 2012). In this study, 15 of 18 microsatellites that were used were 2 bp in length. In addition, the distribution of allele frequencies showed the signature loss of a lower frequency class in selection cutting stands, which indicated a bottlenecked population (Figs 3, S2 and S3).

Recent bottlenecks, which resulted from logging, are typically accompanied by reductions in the mean number of alleles, the number of low‐frequency rare alleles, and allelic richness (Pautasso 2009). In some cases, a reduction in the level of heterozygosity has been observed (Rajora 1999), because allelic diversity is reduced more rapidly than heterozygosity under bottlenecks (Nei et al. 1975; Spencer et al. 2000). We found no differences in *A*_R_, *H*_E_, *H*_O_, and *F*_IS_ between OG and SC stands (Fig. 2). A very low level of differentiation between cohorts in SC stands was detected, while there was no differentiation in OG stands (Table 2). Thus, it appears that SC had a low negative effect on the genetic diversity of the remaining mature sugar maple trees.

The negative impact of SC on sugar maple stands was very weak and could be transient, given that we found (i) a high level of genetic diversity in sugar maple stands, (ii) low genetic differentiation between stands (*F*_ST_ ≤0.004) (Table 1 and Table S7), and (iii) genetic diversity that resided mostly within populations, as revealed by amova (Table 2). Low *F*_ST_ values between stands that were separated by 80 km indicated high levels of gene flow. Long‐distance effective wind dispersal of pollen is reported for trees, for example, up to 100 km for Scots pine (*Pinus sylvestris* L., Kremer et al. 2012). Sugar maple is routinely wind pollinated (Gabriel and Garrett 1984), and its seeds are dispersed to a maximum distance of 100 m (Johnson 1988). Therefore, pollen gene flow is very important in sugar maple populations (e.g., Khodwekar et al. 2015). Low levels of genetic differentiation have been reported for sugar maple stands at local (*F*_ST_ ≤0.017) and regional scales (*F*_ST_ ≤0.049; Ontario to Nova Scotia, Canada; Young et al. 1993a,b).

## Conclusion

In conclusion, our results indicated that high gene flow in both old‐growth and selection cut stands may be sufficient to maintain levels of genetic diversity for future generations. Yet, we also found a negative influence of selection cutting on mature sugar maple diversity, which creates a genetic bottleneck. This result contrast with the generally admitted assumption that selection cut has no impact on forest genetics. Harvesting on our sites had occurred 18 years prior to sampling, and a second selection cut harvest is planned for the same stands. Multiple harvests compound additional losses of genetic diversity, which could possibly lead to erosion of maternal genetic diversity and fixation of deleterious alleles. Selection cutting systems differ from natural microgap disturbance dynamics. These systems did not seem to be fully appropriate for managed sugar maple stands in terms of their effects on genetic diversity as well as other ecological processes (Angers et al. 2005). A number of recommendations were made by Angers et al. (2005) to mitigate the shortage of mature trees that included varying the intensity level of selection cutting by leaving mature trees dispersed within stands or small patches of intact forest within harvested areas. The implementation of various sylvicultural scenarios may help prevent genetic erosion in sugar maple trees. Therefore, we strongly recommend the long‐term monitoring of genetic diversity and ecological structure in sugar maple cohorts after multiple selection cutting, which is essential for developing effective sustainable forest management practices.

## Data archiving statement

Data available from the Dryad Digital Repository: http://dx.doi.org/10.5061/dryad.37354.

## Supporting information


**Figure S1.** Predicted means (95% confidence intervals) of *H*_O_ (a) and *F*_IS_ (b) for cohorts (M: mature sugar maples, Sa: sugar maple saplings, and S: sugar maple seedlings) using markers with < 10 alleles per locus.
**Figure S2.** Allele frequency distributions from old‐growth stands (black bars) and selection cut stands (open bars).
**Figure S3.** Allele frequency distributions from old‐growth stands (black bars) and selection cut stands (open bars).
**Table S1.** Summary of sample site coordinates and protection types.
**Table S2.** Genetic variability estimates of microsatellite markers used in the Québec study of sugar maple (*Acer saccharum*).
**Table S3.** Summary of *P*‐values for Hardy–Weinberg equilibrium using genepop.
**Table S4.** Summary of null allele frequencies for each pair of loci and stands using freena.
**Table S5.** Comparison of mean genetic variability estimates (*A*_R_, *H*_O_, *H*_E_, and *F*_IS_) between old‐growth (OG) and selection cut stands (SC) of sugar maple (*Acer saccharum*) in Québec for pooled individuals (PI), mature trees (M), saplings (Sa), seedlings (S) separately, between cohorts (M, Sa and S) for PI, OG and SC separately, and using all markers and markers with ≤ 10 alleles per locus (A).
**Table S6.** Results of linear mixed‐effects models for genetic variability estimates in Québec, prior to model simplification, and using all markers (*n *=* *324) and markers with ≤ 10 alleles per locus (A; *n *=* *126).
**Table S7.** Pairwise comparisons of population *F*_ST_ (below the diagonal) of pooled individuals, mature trees, saplings and seedlings of sugar maple (*Acer saccharum*) for the 6 stands, in Québec using all markers and markers with ≤ 10 alleles per locus (A). *P*‐values are above the diagonal.
**Table S8.** Genetic variability estimates of sugar maple (*Acer saccharum*) stands in Outaouais, Québec, for mature trees (M), saplings (Sa), seedlings (S) and pooled individuals (PI) using markers with less than ten alleles per locus.
**Table S9.** Number of alleles per frequency class, per cohorts of the stands.
**Table S10.** Bottleneck results based on heterozygosity excess and mode shift, for mature trees (M), saplings (Sa), seedlings (S) and pooled individuals (PI).
