# Supplementary material for: Genetic consequences of selection cutting on sugar maple (Acer saccharum Marshall)
Source: Evol Appl. 2016 May 30;9(6):777–90. doi: 10.1111/eva.12384 (PMC4908464; doi:10.1111/eva.12384)
Supplement: Supplementary file 1 — Figure S1. Predicted means (95% confidence intervals) of H O (a) and F IS (b) for cohorts (M: mature sugar maples, Sa: sugar maple saplings, and S: sugar maple seedlings) using markers with < 10 alleles per locus. Figure S2. Allele frequency distributions from old‐growth stands (black bars) and selection cut stands (open bars). Figure S3. Allele frequency distributions from old‐growth stands (black bars) and selection cut stands (open bars). Table S1. Summary of sample site coordinates and protection types. Table S2. Genetic variability estimates of microsatellite markers used in the Québec study of sugar maple (Acer saccharum). Table S3. Summary of P‐values for Hardy–Weinberg equilibrium using genepop. Table S4. Summary of null allele frequencies for each pair of loci and stands using freena. Table S5. Comparison of mean genetic variability estimates (A R, H O, H E, and F IS) between old‐growth (OG) and selection cut stands (SC) of sugar maple (Acer saccharum) in Québec for pooled individuals (PI), mature trees (M), saplings (Sa), seedlings (S) separately, between cohorts (M, Sa and S) for PI, OG and SC separately, and using all markers and markers with ≤ 10 alleles per locus (A). Table S6. Results of linear mixed‐effects models for genetic variability estimates in Québec, prior to model simplification, and using all markers (n = 324) and markers with ≤ 10 alleles per locus (A; n = 126). Table S7. Pairwise comparisons of population F ST (below the diagonal) of pooled individuals, mature trees, saplings and seedlings of sugar maple (Acer saccharum) for the 6 stands, in Québec using all markers and markers with ≤ 10 alleles per locus (A). P‐values are above the diagonal. Table S8. Genetic variability estimates of sugar maple (Acer saccharum) stands in Outaouais, Québec, for mature trees (M), saplings (Sa), seedlings (S) and pooled individuals (PI) using markers with less than ten alleles per locus. Table S9. Number of alleles per frequency class, per cohorts of the s [file EVA-9-777-s001.doc]

*Evolutionary Applications*

**Supporting Information**

**Genetic consequences of selection cutting on sugar maple (*Acer saccharum* Marshall)**

Noémie Graignic, Francine Tremblay and Yves Bergeron

**Table S1 Summary of sample site coordinates and protection types.**

| Study ID | Study name | Protection type | Latitude | Longitude | Elevation (m) | Distance to paired OG |
| --- | --- | --- | --- | --- | --- | --- |
| OG1 | Lac Tucker | EFE | 45° 56′ 36.6″ N | 75° 47′ 53.5″ W | 386 |  |
| SC1 | Lac St Charles |  | 45° 47′ 37.3″ N | 75° 52′ 03.7″ W | 305 | 17.3 |
| OG2 | Lac de l'Ecluse | EFE | 45° 51′ 35.6″ N | 75° 23′ 55.8″ W | 388 |  |
| SC2 | Lac Faucon |  | 45° 49′ 15.5″ N | 75° 19′ 18.8″ W | 288 | 7.8 |
| OG3 | Lac Marie-Lefranc | EFE | 46° 07′ 05.3″ N | 75° 00′ 36.5″ W | 410 |  |
| SC3 | Lac Marie-Lefranc |  | 46° 05′ 52.8″ N | 75° 00′ 40.3″ W | 386 | 1.6 |

OG, old-growth stand; SC, selection cutting stand; EFE, *écosystèmes forestiers exceptionnels*. For more details on stand characteristics (except for OG2), see Angers et al. (2005), and for OG2 and OG3 (same as 2-C-D and 2-C-C, respectively), see Graignic et al. (2014).

Angers, V. A., C. Messier, M. Beaudet, and A. Leduc 2005. Comparing composition and structure in old-growth and harvested (selection and diameter-limit cuts) northern hardwood stands in Quebec. *Forest Ecology and Management* **217**:275–293.

Graignic, N., F. Tremblay, and Y. Bergeron 2014. Geographical variation in reproductive capacity of sugar maple (*Acer saccharum* Marshall) northern peripheral populations. *Journal of Biogeography* **41**:145–157.

**Table S2** Genetic variability estimates of microsatellite markers used in the Québec study of sugar maple (*Acer saccharum*).

| Locus | GenBank access no. | M | Size range (bp) | *A*_T_ | *A* | *H*_O_ | *H*_E_ | *F*_IS_ | References |
| --- | --- | --- | --- | --- | --- | --- | --- | --- | --- |
| SM11 | KC731552 | 3 | 178–199 | 12 | 8.2 | 0.586 | 0.629 | 0.070 | (Graignic et al. 2013) |
| SM14 | KC751436 | 4 | 70–120 | 21 | 16.2 | 0.742 | 0.894 | 0.170 | (Graignic et al. 2013) |
| SM21A | KC751437 | 4 | 179–237 | 28 | 17.7 | 0.755 | 0.867 | 0.129 | (Graignic et al. 2013) |
| SM22 | KC751438 | 2 | 293–323 | 17 | 13.7 | 0.526 | 0.896 | 0.414 | (Graignic et al. 2013) |
| SM27* | KC751440 | 4 | 242–260 | 9 | 7.0 | 0.403 | 0.673 | 0.405 | (Graignic et al. 2013) |
| SM29* | KC751441 | 4 | 278–307 | 10 | 7.5 | 0.527 | 0.724 | 0.274 | (Graignic et al. 2013) |
| SM34 | KC751442 | 3 | 118–167 | 23 | 15.8 | 0.772 | 0.843 | 0.084 | (Graignic et al. 2013) |
| SM36 | KC751443 | 5 | 146–182 | 18 | 13.3 | 0.714 | 0.828 | 0.137 | (Graignic et al. 2013) |
| SM37 | KC751444 | 2 | 174–196 | 12 | 9.0 | 0.564 | 0.667 | 0.153 | (Graignic et al. 2013) |
| SM42 | KC751445 | 1 | 90–133 | 17 | 9.5 | 0.678 | 0.804 | 0.157 | (Graignic et al. 2013) |
| SM47 | KC751446 | 2 | 201–225 | 12 | 8.2 | 0.390 | 0.667 | 0.415 | (Graignic et al. 2013) |
| SM51* | KC751447 | 1 | 269–290 | 7 | 5.5 | 0.344 | 0.464 | 0.258 | (Graignic et al. 2013) |
| SM53* | KC751448 | 5 | 287–310 | 7 | 3.0 | 0.352 | 0.517 | 0.317 | (Graignic et al. 2013) |
| SM55 | KC751449 | 2 | 248–276 | 15 | 10.3 | 0.480 | 0.686 | 0.296 | (Graignic et al. 2013) |
| SM56* | KC751450 | 3 | 287–299 | 6 | 5.0 | 0.364 | 0.611 | 0.400 | (Graignic et al. 2013) |
| SM60* | KC751452 | 3 | 231–237 | 3 | 2.8 | 0.305 | 0.410 | 0.246 | (Graignic et al. 2013) |
| Aop943* | EF531298 | 1 | 143–160 | 8 | 5.7 | 0.565 | 0.567 | 0.002 | (Segarra-Moragues et al. 2008)  (Graignic et al. 2013) |
| Am116 | AB303350 | 1 | 230–267 | 18 | 11.5 | 0.589 | 0.701 | 0.160 | (Kikuchi and Shibata 2008)  (Graignic et al. 2013) |

M, multiplexing arrangement; *A*_T_, total number of alleles; *A*, mean number of alleles per locus; *H*_O_, mean observed heterozygosity; *H*_E_, mean expected heterozygosity; *F*_IS_, inbreeding coefficient. * indicates markers with ≤ 10 alleles per locus.

Graignic, N., F. Tremblay, and Y. Bergeron 2013. Development of polymorphic nuclear microsatellite markers in sugar maple (*Acer saccharum* Marsh.) using cross-species transfer and SSR-enriched shotgun pyrosequencing. *Conservation Genetics Resources* **5**:845–848.

Kikuchi, S. and M. Shibata 2008. Development of polymorphic microsatellite markers in *Acer mono* Maxim. *Molecular Ecology Notes* **8**:339–341.

Segarra-Moragues, J., G. Gleiser, and F. González-Candelas 2008. Isolation and characterization of microsatellite loci in *Acer opalus* (Aceraceae), a sexually-polymorphic tree, through an enriched genomic library. *Conservation Genetics* **9**:1059–1062.

**Table S3** Summary of *P*-values for Hardy–Weinberg equilibrium using genepop.

|  | OG1 | SC1 | OG2 | SC2 | OG3 | SC3 |
| --- | --- | --- | --- | --- | --- | --- |
| SM11 | 0.1903 | 0.1722 | 0.5947 | 0.0212 | 0.1487 | 0.0110 |
| SM14 | 0.0011 | 0.0026 | 0.0102 | 0.0027 | 0.0035 | **0.0000** |
| SM21A | 0.0384 | 0.1047 | 0.0140 | 0.0066 | 0.8285 | 0.0370 |
| SM22* | **0.0000** | **0.0000** | **0.0000** | **0.0000** | **0.0000** | **0.0000** |
| SM27* | **0.0002** | **0.0000** | **0.0000** | 0.0017 | 0.0028 | **0.0000** |
| SM29 | **0.0004** | 0.0024 | 0.0072 | 0.0052 | 0.0735 | 0.0077 |
| SM34 | 0.0163 | 0.1669 | 0.1153 | 0.3337 | 0.3417 | 0.2668 |
| SM36 | 0.1236 | 0.0788 | 0.5338 | 0.1049 | 0.2210 | **0.0000** |
| SM37 | 0.7559 | 0.0632 | 0.0316 | 0.0175 | 0.3030 | 0.0013 |
| SM42 | 0.2747 | 0.0067 | **0.0000** | 0.3748 | 0.0674 | 0.0007 |
| SM47* | **0.0000** | **0.0000** | **0.0000** | **0.0000** | **0.0000** | **0.0001** |
| SM51 | 0.0328 | 0.1688 | 0.0963 | 0.0050 | 0.2176 | 0.0583 |
| SM53 | **0.0000** | 0.5993 | 0.0048 | 0.4135 | 0.0008 | 0.0007 |
| SM55* | **0.0000** | 0.0541 | 0.0027 | **0.0000** | 0.0023 | **0.0001** |
| SM56* | 0.0039 | **0.0000** | **0.0000** | **0.0001** | **0.0000** | 0.2673 |
| SM60 | 0.5995 | **0.0000** | 0.0519 | 0.0959 | 0.0025 | 0.2962 |
| Aop943 | 0.0979 | 0.8544 | 0.0638 | 0.7918 | 0.7999 | 0.0339 |
| Am116 | **0.0000** | 0.0189 | 0.5458 | **0.0000** | 0.5295 | 0.1456 |

Markov chain parameters: 10 000 dememorizations, followed by 500 batches of 5 000 iterations per batch. *P*-values significant after Bonferroni correction are in bold type. * indicates locus at which most stands show signs of deviance from HWE.

**Table S4** Summary of null allele frequencies for each pair of loci and stands using freena.

|  | OG1 | SC1 | OG2 | SC2 | OG3 | SC3 |
| --- | --- | --- | --- | --- | --- | --- |
| SM11 | 0.068 | 0.043 | 0.022 | 0.076 | 0.036 | 0.005 |
| SM14 | 0.061 | 0.088 | 0.058 | 0.066 | 0.067 | **0.110** |
| SM21A | 0.066 | 0.073 | 0.086 | 0.080 | 0.000 | 0.041 |
| SM22* | **0.248** | **0.212** | **0.182** | **0.185** | **0.151** | **0.165** |
| SM27* | **0.141** | **0.197** | **0.186** | **0.111** | **0.127** | **0.220** |
| SM29* | **0.142** | **0.121** | **0.112** | **0.128** | 0.071 | **0.116** |
| SM34 | 0.092 | 0.010 | 0.005 | 0.022 | 0.000 | 0.036 |
| SM36 | 0.047 | 0.039 | 0.013 | 0.032 | 0.040 | **0.147** |
| SM37 | 0.000 | 0.058 | 0.074 | 0.037 | 0.000 | **0.132** |
| SM42 | 0.031 | 0.034 | 0.070 | 0.035 | 0.063 | **0.104** |
| SM47* | **0.158** | **0.145** | **0.202** | **0.203** | **0.104** | **0.164** |
| SM51 | 0.075 | 0.082 | 0.092 | **0.106** | 0.065 | 0.096 |
| SM53* | **0.184** | 0.023 | **0.125** | 0.038 | **0.139** | **0.128** |
| SM55* | **0.168** | **0.108** | 0.034 | **0.153** | **0.105** | **0.138** |
| SM56* | **0.133** | **0.185** | **0.185** | **0.170** | **0.205** | 0.067 |
| SM60 | 0.013 | **0.150** | 0.065 | 0.075 | **0.134** | 0.045 |
| Aop943 | 0.061 | 0.006 | 0.000 | 0.000 | 0.000 | 0.061 |
| Am116 | **0.155** | 0.087 | 0.000 | **0.164** | 0.000 | 0.083 |

High (≥ 10 %) frequencies of null alleles are in bold type. * indicates locus at which most stands show signs of null alleles.

**Table S5** Comparison of mean genetic variability estimates (*A*_R_, *H*_O_, *H*_E_, and *F*_IS_) between old-growth (OG) and selection cut stands (SC) of sugar maple (*Acer saccharum*) in Québec for pooled individuals (PI), mature trees (M), saplings (Sa), seedlings (S) separately, between cohorts (M, Sa and S) for PI, OG and SC separately, and using all markers and markers with ≤ 10 alleles per locus (A).

| Markers | Genetic Indices | Data | OG | SC | *P*-value | Data | M | Sa | S | *P*-value |
| --- | --- | --- | --- | --- | --- | --- | --- | --- | --- | --- |
| All markers | *A*_R_ | PI | 9.011 | 8.983 | 0.9880 | PI | 6.288 | 6.227 | 6.299 | 0.8370 |
|  | *H*_O_ |  | 0.546 | 0.528 | 0.5030 |  | 0.594 | 0.503 | 0.512 | **0.0040** |
|  | *H*_E_ |  | 0.689 | 0.694 | 0.1960 |  | 0.696 | 0.682 | 0.691 | 0.1050 |
|  | *F*_IS_ |  | 0.208 | 0.240 | 0.4040 |  | 0.146 | 0.262 | 0.260 | **0.0030** |
|  | *A*_R_ | M | 6.557 | 6.310 | 0.2900 | OG | 6.401 | 6.131 | 6.352 | 0.2260 |
|  | *H*_O_ |  | 0.610 | 0.577 | 0.1780 |  | 0.610 | 0.502 | 0.519 | **0.0150** |
|  | *H*_E_ |  | 0.694 | 0.698 | 0.6100 |  | 0.694 | 0.679 | 0.688 | 0.3500 |
|  | *F*_IS_ |  | 0.121 | 0.173 | 0.1780 |  | 0.121 | 0.261 | 0.245 | **0.0190** |
|  | *A*_R_ | Sa | 6.131 | 6.323 | 0.5330 | SC | 6.175 | 6.323 | 6.245 | 0.7090 |
|  | *H*_O_ |  | 0.502 | 0.503 | 1.0000 |  | 0.577 | 0.503 | 0.504 | 0.1750 |
|  | *H*_E_ |  | 0.679 | 0.684 | 0.8140 |  | 0.698 | 0.684 | 0.695 | 0.3890 |
|  | *F*_IS_ |  | 0.261 | 0.264 | 0.8320 |  | 0.173 | 0.264 | 0.275 | 0.1780 |
|  | *A*_R_ | S | 6.352 | 6.245 | 0.4170 |  |  |  |  |  |
|  | *H*_O_ |  | 0.519 | 0.504 | 0.7200 |  |  |  |  |  |
|  | *H*_E_ |  | 0.688 | 0.695 | 0.7080 |  |  |  |  |  |
|  | *F*_IS_ |  | 0.245 | 0.275 | 0.6200 |  |  |  |  |  |
| Markers A | *A*_R_ | PI | 3.995 | 4.030 | 0.7320 | PI | 3.902 | 4.079 | 4.056 | 0.2960 |
|  | *H*_O_ |  | 0.408 | 0.408 | 0.9970 |  | 0.487 | 0.365 | 0.370 | **0.0010** |
|  | *H*_E_ |  | 0.566 | 0.562 | 0.7770 |  | 0.573 | 0.551 | 0.570 | 0.4130 |
|  | *F*_IS_ |  | 0.279 | 0.274 | 0.9290 |  | 0.149 | 0.336 | 0.350 | **0.0010** |
|  | *A*_R_ | M | 3.886 | 3.918 | 0.8660 | OG | 3.886 | 4.165 | 3.933 | 0.2550 |
|  | *H*_O_ |  | 0.491 | 0.484 | 0.8900 |  | 0.491 | 0.358 | 0.371 | **0.0130** |
|  | *H*_E_ |  | 0.569 | 0.576 | 0.7990 |  | 0.569 | 0.567 | 0.562 | 0.9490 |
|  | *F*_IS_ |  | 0.137 | 0.160 | 0.7970 |  | 0.137 | 0.368 | 0.340 | **0.0090** |
|  | *A*_R_ | Sa | 4.165 | 3.993 | 0.3420 | SC | 3.918 | 3.993 | 4.178 | 0.3350 |
|  | *H*_O_ |  | 0.358 | 0.372 | 0.8180 |  | 0.484 | 0.372 | 0.370 | *0.0590* |
|  | *H*_E_ |  | 0.567 | 0.534 | 0.1930 |  | 0.576 | 0.534 | 0.577 | *0.0960* |
|  | *F*_IS_ |  | 0.368 | 0.304 | 0.4860 |  | 0.160 | 0.304 | 0.359 | *0.0760* |
|  | *A*_R_ | S | 3.933 | 4.178 | 0.1770 |  |  |  |  |  |
|  | *H*_O_ |  | 0.371 | 0.370 | 0.9910 |  |  |  |  |  |
|  | *H*_E_ |  | 0.562 | 0.577 | 0.5550 |  |  |  |  |  |
|  | *F*_IS_ |  | 0.340 | 0.359 | 0.8270 |  |  |  |  |  |

Analyses were performed using fstat and tested for significance using 1000 permutations. *A*_R_, mean allelic richness; *H*_O_, mean observed heterozygosity; *H*_E_, mean expected heterozygosity; *F*_IS_, inbreeding coefficient. Significant values (α = 0.05) are in bold type.

**Table S6** Results of linear mixed-effects models for genetic variability estimates in Québec, prior to model simplification, and using all markers (*n* = 324) and markers with ≤ 10 alleles per locus (A; *n* = 126).

| Markers | Response variable | Explanatory variables | Num. *df* | Denom. *d.f.* | *F* | *P* |
| --- | --- | --- | --- | --- | --- | --- |
| All markers | *A*_R_ | forest type | 1 | 301 | 0.179 | 0.6729 |
|  |  | cohort | 2 | 301 | 0.165 | 0.8483 |
|  |  | forest type × cohort | 2 | 301 | 1.261 | 0.2850 |
|  | *H*_O_ | forest type | 1 | 301 | 1.407 | 0.2364 |
|  |  | cohort | 2 | 301 | 19.413 | **< 0.001** |
|  |  | forest type × cohort | 2 | 301 | 0.535 | 0.5865 |
|  | *H*_E_ | forest type | 1 | 301 | 0.436 | 0.5096 |
|  |  | cohort | 2 | 301 | 1.246 | 0.2891 |
|  |  | forest type × cohort | 2 | 301 | 0.024 | 0.9767 |
|  | *F*_IS_ | forest type | 1 | 301 | 1.880 | 0.1713 |
|  |  | cohort | 2 | 301 | 17.258 | **< 0.0001** |
|  |  | forest type × cohort | 2 | 301 | 0.657 | 0.5194 |
| Markers A | *A*_R_ | forest type | 1 | 114 | 0.053 | 0.8187 |
|  |  | cohort | 2 | 114 | 0.791 | 0.4560 |
|  |  | forest type × cohort | 2 | 114 | 0.870 | 0.4218 |
|  | *H*_O_ | forest type | 1 | 114 | 0.010 | 0.9207 |
|  |  | cohort | 2 | 114 | 14.081 | **< 0.0001** |
|  |  | forest type × cohort | 2 | 114 | 0.072 | 0.9305 |
|  | *H*_E_ | forest type | 1 | 114 | 0.068 | 0.7947 |
|  |  | cohort | 2 | 114 | 0.811 | 0.4470 |
|  |  | forest type × cohort | 2 | 114 | 0.909 | 0.4058 |
|  | *F*_IS_ | forest type | 1 | 114 | 0.097 | 0.7557 |
|  |  | cohort | 2 | 114 | 12.391 | **< 0.0001** |
|  |  | forest type × cohort | 2 | 114 | 0.688 | 0.5047 |

Num. *d.f.,* numerator degrees-of-freedom; denom. *d.f.*, denominator degrees-of-freedom; *A*_R_, mean allelic richness; *H*_O_, mean observed heterozygosity; *H*_E_, mean expected heterozygosity; *F*_IS_, inbreeding coefficient. Significant values at α = 0.05 are in bold type and at α = 0.10 in italics.

**Table S7** Pairwise comparisons of population *F*_ST_ (below the diagonal) of pooled individuals, mature trees, saplings and seedlings of sugar maple (*Acer saccharum*) for the 6 stands, in Québec using all markers and markers with ≤ 10 alleles per locus (A). *P*-values are above the diagonal.

|  | OG1 | SC1 | OG2 | SC2 | OG3 | SC3 |
| --- | --- | --- | --- | --- | --- | --- |
| All markers | | | | | | |
| Pooled individuals | | | | | | |
| OG1 | — | 0.58000 | 0.57667 | 0.22333 | 0.45667 | 0.11000 |
| SC1 | -0.0001 | — | 0.89333 | 0.57667 | **0.00333** | **0.00333** |
| OG2 | -0.0014 | -0.0016 | — | 0.31667 | 0.10000 | 0.04333 |
| SC2 | 0.0015 | -0.0005 | -0.0010 | — | 0.03000 | 0.02333 |
| OG3 | 0.0014 | **0.0039** | -0.0002 | 0.0008 | — | **0.00333** |
| SC3 | -0.0020 | **0.0021** | 0.0000 | 0.0009 | **0.0044** | — |
| Mature trees | | | | | | |
| OG1 | — | 0.07667 | 0.01667 | 0.02667 | 0.03333 | 0.56000 |
| SC1 | 0.0041 | — | 0.27333 | 0.14667 | 0.05333 | 0.16333 |
| OG2 | 0.0078 | 0.0005 | — | 0.01667 | 0.51333 | 0.05000 |
| SC2 | 0.0077 | -0.0003 | 0.0061 | — | 0.02000 | 0.14000 |
| OG3 | 0.0040 | 0.0022 | -0.0056 | 0.0069 | — | 0.02000 |
| SC3 | -00053 | -0.0009 | -0.0002 | 0.0084 | 0.0031 | — |
| Saplings |  |  |  |  |  |  |
| OG1 | — | 0.06000 | 0.16667 | 0.28333 | 0.49333 | 0.18667 |
| SC1 | 0.0065 | — | 0.21333 | 0.14667 | 0.18667 | 0.17333 |
| OG2 | -0.0022 | 0.0059 | — | 0.28000 | 0.32667 | 0.50000 |
| SC2 | -0.0044 | 0.0113 | -0.0013 | — | 0.08333 | 0.04000 |
| OG3 | -0.0007 | 0.0071 | 0.0132 | 0.0051 | — | 0.27000 |
| SC3 | -0.0019 | 0.0059 | -0.0006 | 0.0065 | 0.0073 | — |
| Seedlings | | | | | | |
| OG1 | — | 0.19667 | 0.21667 | 0.20000 | 0.17000 | 0.07667 |
| SC1 | 0.0019 | — | 0.89333 | 0.75000 | 0.01667 | 0.19333 |
| OG2 | -0.0035 | -0.0053 | — | 0.03333 | 0.06667 | 0.37333 |
| SC2 | 0.0155 | 0.0034 | 0.0118 | — | 0.15000 | 0.18333 |
| OG3 | 0.0051 | 0.0063 | 0.0051 | 0.0079 | — | 0.01000 |
| SC3 | 0.0071 | 0.0135 | 0.0013 | 0.0063 | 0.0209 | — |
| Markers A | |  |  |  |  |  |
| Pooled individuals |  |  |  |  |  |  |
| OG1 | — | 0.19667 | 0.60333 | 0.31000 | 0.11667 | 0.73000 |
| SC1 | 0.0017 | — | 0.77667 | 0.52000 | 0.04333 | 0.01667 |
| OG2 | 0.0005 | -0.0035 | — | 0.93667 | 0.07333 | 0.44667 |
| SC2 | 0.0018 | 0.0011 | -0.0047 | — | 0.06000 | 0.47333 |
| OG3 | 0.0055 | 0.0087 | 0.0027 | 0.0052 | — | **0.00333** |
| SC3 | -0.0047 | 0.0083 | -0.0009 | -0.0013 | **0.0123** | — |
| Mature trees |  |  |  |  |  |  |
| OG1 | — | 0.09667 | 0.05667 | 0.08667 | 0.11667 | 0.04333 |
| SC1 | 0.0048 | — | 0.61000 | 0.24000 | 0.01000 | 0.21333 |
| OG2 | 0.0185 | 0.0002 | — | 0.25000 | 0.11000 | 0.70000 |
| SC2 | 0.0158 | -0.0076 | 0.0049 | — | 0.05000 | 0.27000 |
| OG3 | 0.0040 | 0.0148 | 0.0039 | 0.0072 | — | 0.01000 |
| SC3 | -0.0003 | 0.0042 | -0.0030 | 0.0120 | 0.0150 | — |
| Saplings |  |  |  |  |  |  |
| OG1 | — | 0.06000 | 0.53333 | 0.83000 | 0.37000 | 0.77667 |
| SC1 | 0.0155 | — | 0.13000 | 0.29000 | 0.01000 | 0.04000 |
| OG2 | 0.0102 | 0.0115 | — | 0.95000 | 0.16667 | 0.70667 |
| SC2 | -0.0041 | 0.0163 | -0.0033 | — | 0.13333 | 0.48000 |
| OG3 | 0.0028 | 0.0290 | 0.0264 | 0.0114 | — | 0.01667 |
| SC3 | -0.0069 | 0.0239 | -0.0106 | -0.0042 | 0.0245 | — |
| Seedlings |  |  |  |  |  |  |
| OG1 | — | 0.68333 | 0.42000 | 0.49333 | 0.76000 | 0.19000 |
| SC1 | -0.0143 | — | 0.94333 | 0.28667 | 0.43333 | 0.05667 |
| OG2 | -0.0090 | -0.0208 | — | 0.35000 | 0.15000 | 0.56000 |
| SC2 | 0.0099 | 0.0061 | -0.0059 | — | 0.19667 | 0.22000 |
| OG3 | -0.0104 | -0.0059 | 0.0037 | 0.0096 | — | 0.03000 |
| SC3 | 0.0161 | 0.0289 | -0.0062 | 0.0090 | 0.0301 | — |

Significant values after adjusted nominal level (α = 0.05) for multiple comparisons are in bold type.

**Table S8** Genetic variability estimates of sugar maple (*Acer saccharum*) stands in Outaouais, Québec, for mature trees (M), saplings (Sa), seedlings (S) and pooled individuals (PI) using markers with less than ten alleles per locus.

| Stands | Cohorts | N | *A* | *A*_R_ | *A*_R_^*^ | *H*_O_ | *H*_E_ | *F*_IS_ | *F*_ST_ |
| --- | --- | --- | --- | --- | --- | --- | --- | --- | --- |
| OG1 | M | 20 | 4.3 | — | 4.1 | 0.487 | 0.567 | 0.140 | 0.005 |
|  | Sa | 20 | 4.1 | — | 4.0 | 0.295 | 0.554 | 0.468*** | 0.002 |
|  | S | 20 | 4.0 | — | 3.9 | 0.369 | 0.527 | 0.300*** | 0.002 |
|  | PI | 60 | 5.1 | 5.0 | — | 0.384 | 0.550 | 0.302*** | 0.001 |
| SC1 | M | 20 | 3.9 | — | 3.7 | 0.461 | 0.571 | 0.191** | 0.003 |
|  | Sa | 20 | 3.7 | — | 3.6 | 0.355 | 0.587 | 0.396*** | 0.011 |
|  | S | 20 | 4.6 | — | 4.3 | 0.372 | 0.560 | 0.336*** | 0.001 |
|  | PI | 60 | 5.3 | 5.1 | — | 0.397 | 0.574 | 0.308*** | 0.003 |
| OG2 | M | 20 | 4.1 | — | 3.9 | 0.499 | 0.583 | 0.144*** | 0.005 |
|  | Sa | 20 | 4.4 | — | 4.3 | 0.390 | 0.571 | 0.318*** | 0.000 |
|  | S | 20 | 4.1 | — | 4.0 | 0.344 | 0.566 | 0.392*** | -0.009 |
|  | PI | 60 | 5.1 | 5.0 | — | 0.412 | 0.572 | 0.280*** | -0.001 |
| SC2 | M | 20 | 4.3 | — | 4.2 | 0.548 | 0.597 | 0.081 | 0.006 |
|  | Sa | 20 | 4.4 | — | 4.2 | 0.360 | 0.503 | 0.283*** | 0.005 |
|  | S | 20 | 4.4 | — | 4.2 | 0.427 | 0.626 | 0.319*** | 0.003 |
|  | PI | 60 | 5.6 | 5.4 | — | 0.440 | 0.577 | 0.236*** | 0.000 |
| OG3 | M | 22 | 3.9 | — | 3.6 | 0.491 | 0.563 | 0.128 | 0.007 |
|  | Sa | 20 | 4.4 | — | 4.2 | 0.392 | 0.580 | 0.323*** | 0.011 |
|  | S | 18 | 4.0 | — | 3.9 | 0.402 | 0.598 | 0.327*** | 0.010 |
|  | PI | 60 | 5.0 | 4.8 | — | 0.430 | 0.581 | 0.260*** | 0.006 |
| SC3 | M | 20 | 4.0 | — | 3.8 | 0.449 | 0.564 | 0.204*** | 0.001 |
|  | Sa | 20 | 4.4 | — | 4.2 | 0.401 | 0.515 | 0.221*** | 0.007 |
|  | S | 20 | 4.3 | — | 4.0 | 0.315 | 0.549 | 0.427*** | 0.008 |
|  | PI | 60 | 5.1 | 5.0 | — | 0.388 | 0.545 | 0.288*** | 0.002 |
| Means |  | 60 | 5.2 | 5.1 | 4.0 | 0.408 | 0.571 | 0.279*** | 0.002 |
| All |  | 360 | 7.1 | 7.1 |  | 0.408 | 0.567 | 0.280*** |  |

To compare allelic richness among cohorts (M, Sa, S), we calculated allelic richness using those cohorts in the same database (*A*_R_^*^). Populations were old-growth (OG) forest or had received a single selection cutting (SC) at the end of 1990–beginning of 1991. N, number of individuals; *A*, mean number of alleles per locus; *A*_R_, mean allelic richness; *H*_O_, mean observed heterozygosity; *H*_E_, mean expected heterozygosity; *F*_IS_, inbreeding coefficient; *F*_ST_, mean pairwise *F*_ST_; Means were determined using PI except for *A*_R_^*^. *** *P* ≤ 0.001; ** 0.001 < *P* ≤ 0.005; * 0.005 < *P* ≤ 0.010 for tests of heterozygote deficiency.

**
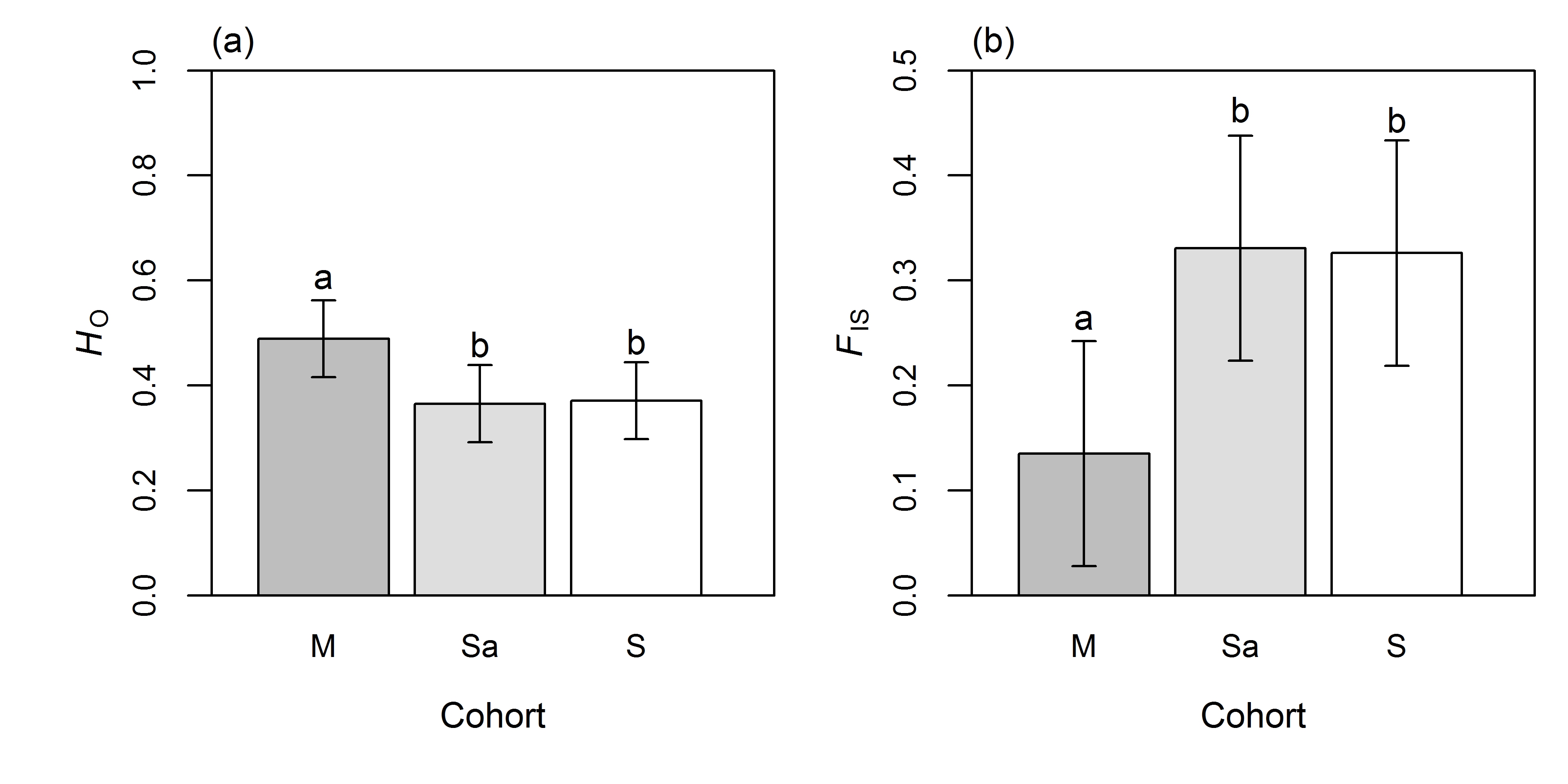
**

**Figure S1** Predicted means (95% confidence intervals) of *H*_O_ (a) and *F*_IS_ (b) for cohorts (M: mature sugar maples, Sa: sugar maple saplings, and S: sugar maple seedlings) using markers with < 10 alleles per locus. *H*_O_, mean observed heterozygosity; *F*_IS_, inbreeding coefficient. Means with the same letter do not differ at α = 0.05, but differ (with an asterisk) at α = 0.10.

**Table S9** Number of alleles per frequency class, per cohorts of the stands.

| Stands | Cohorts | *A*_T_ | C | I | L0.01 | R0.01 | L0.05 | R0.05 |
| --- | --- | --- | --- | --- | --- | --- | --- | --- |
| OG1 | M | 128 | 2 (2%) | 20 (16%) | 106 (83%) | 0 (0%) | 64 (50%) | 42 (33%) |
|  | Sa | 119 | 1 (1%) | 26 (22%) | 92 (77%) | 0 (0%) | 54 (45%) | 38 (32%) |
|  | S | 128 | 1 (1%) | 22 (17%) | 105 (82%) | 0 (0%) | 67 (52%) | 38 (30%) |
|  | PI | 169 | 1 (1%) | 18 (11%) | 122 (72%) | 28 (17%) | 58 (34%) | 92 (54%) |
| SC1 | M | 129 | 0 (0%) | 24 (19%) | 105 (81%) | 0 (0%) | 66 (51%) | 39 (30%) |
|  | Sa | 124 | 1 (1%) | 23 (19%) | 100 (81%) | 0 (0%) | 59 (48%) | 41 (33%) |
|  | S | 124 | 1 (1%) | 20 (16%) | 103 (83%) | 0 (0%) | 64 (52%) | 39 (31%) |
|  | PI | 171 | 0 (0%) | 22 (13%) | 116 (68%) | 33 (19%) | 58 (34%) | 91 (53%) |
| OG2 | M | 125 | 0 (0%) | 22 (18%) | 103 (82%) | 0 (0%) | 64 (51%) | 39 (31%) |
|  | Sa | 125 | 1 (1%) | 23 (18%) | 101 (81%) | 0 (0%) | 66 (53%) | 35 (28%) |
|  | S | 126 | 0 (0%) | 22 (17%) | 104 (83%) | 0 (0%) | 62 (49%) | 42 (33%) |
|  | PI | 171 | 0 (0%) | 23 (13%) | 114 (67%) | 34 (20%) | 55 (32%) | 93 (54%) |
| SC2 | M | 117 | 1 (1%) | 25 (21%) | 91 (78%) | 0 (0%) | 63 (54%) | 28 (24%) |
|  | Sa | 120 | 2 (2%) | 20 (17%) | 98 (82%) | 0 (0%) | 58 (48%) | 40 (33%) |
|  | S | 126 | 0 (0%) | 23 (18%) | 103 (82%) | 0 (0%) | 64 (51%) | 39 (31%) |
|  | PI | 171 | 0 (0%) | 22 (13%) | 113 (66%) | 36 (21%) | 51 (30%) | 98 (57%) |
| OG3 | M | 137 | 1 (1%) | 21 (15%) | 115 (84%) | 0 (0%) | 48 (35%) | 67 (49%) |
|  | Sa | 121 | 1 (1%) | 24 (20%) | 96 (79%) | 0 (0%) | 58 (48%) | 38 (31%) |
|  | S | 123 | 1 (1%) | 21 (17%) | 101 (82%) | 0 (0%) | 60 (49%) | 41 (33%) |
|  | PI | 171 | 0 (0%) | 22 (13%) | 113 (66%) | 36 (21%) | 52 (30%) | 97 (57%) |
| SC3 | M | 117 | 1 (1%) | 21 (18%) | 95 (81%) | 0 (0%) | 58 (50%) | 37 (32%) |
|  | Sa | 134 | 2 (1%) | 18 (13%) | 114 (85%) | 0 (0%) | 73 (54%) | 41 (31%) |
|  | S | 122 | 1 (1%) | 24 (20%) | 97 (80%) | 0 (0%) | 57 (47%) | 40 (33%) |
|  | PI | 166 | 1 (1%) | 18 (11%) | 115 (69%) | 32 (19%) | 59 (36%) | 88 (53%) |

*A*_T_, total number of alleles; C, common *f* ≥ 0.75; I, intermediate 0.75 > *f* ≥ 0.25; L0.01, low 0.25 > *f* ≥ 0.01; R0.01, rare *f* < 0.01; L0.05, low 0.25 > *f* ≥ 0.05; R0.05, rare *f* < 0.05; M, mature sugar maples; Sa, sugar maple saplings; S, sugar maple seedlings; PI, pooled individuals.

**Table S10** Bottleneck results based on heterozygosity excess and mode shift, for mature trees (M), saplings (Sa), seedlings (S) and pooled individuals (PI).

|  |  | Heterozygosity excess | | | | | | Mode |
| --- | --- | --- | --- | --- | --- | --- | --- | --- |
| Stands | Cohorts | IAM | TMM | | | | SMM | shift |
|  |  |  | 70% | 90% | 95% | 99% |  |  |
| OG1 | M | 0.07076 | 0.97586 | 0.99398 | 0.99832 | 0.99903 | 0.99883 | Normal |
|  | Sa | 0.07702 | 0.98288 | 0.99832 | 0.99961 | 0.99983 | 0.99979 | Normal |
|  | S | 0.18461 | 0.93513 | 0.98959 | 0.99088 | 0.99763 | 0.99763 | Normal |
|  | PI | 0.08368 | 0.97003 | 0.99961 | 0.99994 | 1.00000 | 1.00000 | Normal |
| SC1 | M | **0.00200** | 0.79144 | 0.96673 | 0.97586 | 0.99088 | 0.99203 | Normal |
|  | Sa | 0.07702 | 0.83764 | 0.96673 | 0.97307 | 0.98075 | 0.98288 | Normal |
|  | S | 0.17327 | 0.91632 | 0.98482 | 0.98959 | 0.99398 | 0.99480 | Normal |
|  | PI | **0.04488** | 0.98075 | 0.99671 | 0.99883 | 0.99968 | 0.99974 | Normal |
| OG2 | M | **0.04488** | 0.73869 | 0.91632 | 0.95929 | 0.97842 | 0.98658 | Normal |
|  | Sa | 0.14186 | 0.90181 | 0.99860 | 0.99961 | 0.99990 | 0.99992 | Normal |
|  | S | **0.02693** | 0.81539 | 0.93513 | 0.97586 | 0.98482 | 0.98816 | Normal |
|  | PI | 0.06487 | 0.98658 | 0.99968 | 0.99992 | 0.99999 | 0.99999 | Normal |
| SC2 | M | **0.00694** | 0.73869 | 0.93513 | 0.95929 | 0.98288 | 0.98816 | Normal |
|  | Sa | 0.22115 | 0.97586 | 0.99306 | 0.99860 | 0.99883 | 0.99883 | Normal |
|  | S | 0.06487 | 0.81539 | 0.97307 | 0.97586 | 0.98816 | 0.98959 | Normal |
|  | PI | **0.04071** | 0.99552 | 0.99983 | 0.99995 | 0.99995 | 0.99997 | Normal |
| OG3 | M | 0.05935 | 0.94065 | 0.99088 | 0.99398 | 0.99763 | 0.99832 | Normal |
|  | Sa | 0.13226 | 0.87690 | 0.97586 | 0.98288 | 0.98816 | 0.99203 | Normal |
|  | S | 0.17327 | 0.85814 | 0.97003 | 0.98816 | 0.99306 | 0.99306 | Normal |
|  | PI | 0.05935 | 0.97842 | 0.99480 | 0.99832 | 0.99903 | 0.99961 | Normal |
| SC3 | M | **0.01518** | 0.61699 | 0.88562 | 0.94581 | 0.97307 | 0.97842 | Normal |
|  | Sa | **0.01925** | 0.97586 | 0.99832 | 0.99883 | 0.99994 | 0.99994 | Normal |
|  | S | 0.09819 | 0.90181 | 0.99203 | 0.99671 | 0.99763 | 0.99800 | Normal |
|  | PI | **0.00140** | 0.99800 | 0.99995 | 0.99999 | 1.00000 | 1.00000 | Normal |

Significant values (α = 0.05) are in bold type.


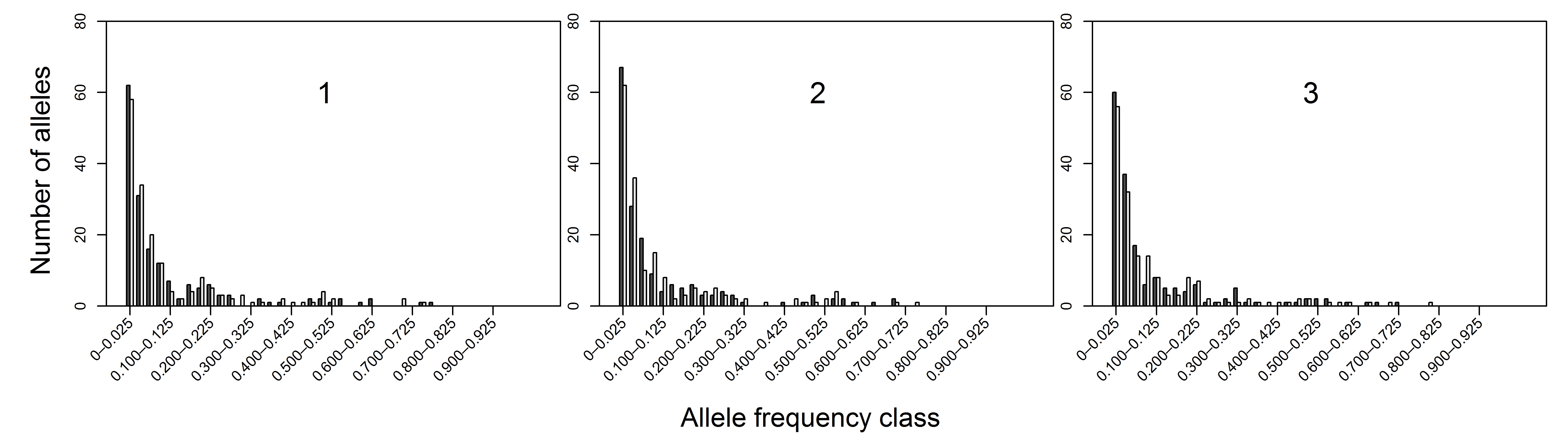


**Figure S2** Allele frequency distributions from old-growth stands (black bars) and selection cut stands (open bars). 1, OG1 and SC1; 2, OG2 and SC2, and 3, OG3 and SC3.


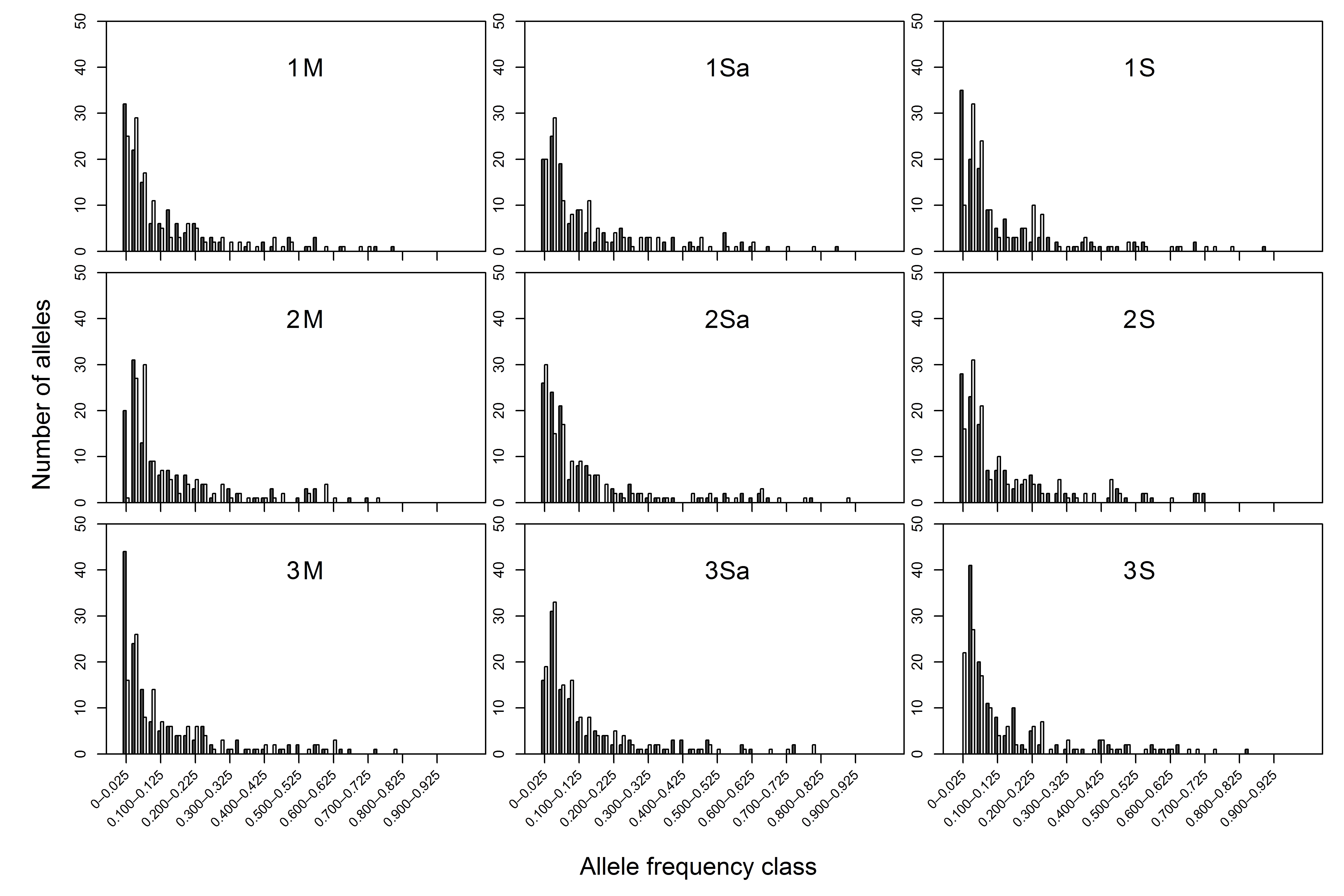


**Figure S3** Allele frequency distributions from old-growth stands (black bars) and selection cut stands (open bars). 1M, 1Sa and 1S, mature sugar maples, saplings and seedlings from OG1 and SC1; 2M, 2Sa and 2S, mature sugar maples, saplings and seedlings from OG2 and SC2, and 3M, 3Sa and 3S, mature sugar maples, saplings and seedlings from OG3 and SC3.
